# DFT-aided machine learning-based discovery of magnetism in Fe-based bimetallic chalcogenides

**DOI:** 10.1038/s41598-023-30438-w

**Published:** 2023-02-25

**Authors:** Dharmendra Pant, Suresh Pokharel, Subhasish Mandal, Dukka B. KC, Ranjit Pati

**Affiliations:** 1 Department of Physics, Michigan Technological University, Houghton, MI 49931 USA; 2 Department of Computer Science, Michigan Technological University, Houghton, MI 49931 USA; 3 Department of Physics and Astronomy, West Virginia University, Morgantown, WV 26506 USA; 4 Henes Center for Quantum Phenomena, Michigan Technological University, Houghton, MI 49931 USA

**Keywords:** Magnetic properties and materials, Scientific data

## Abstract

With the technological advancement in recent years and the widespread use of magnetism in every sector of the current technology, a search for a low-cost magnetic material has been more important than ever. The discovery of magnetism in alternate materials such as metal chalcogenides with abundant atomic constituents would be a milestone in such a scenario. However, considering the multitude of possible chalcogenide configurations, predictive computational modeling or experimental synthesis is an open challenge. Here, we recourse to a stacked generalization machine learning model to predict magnetic moment (µB) in hexagonal Fe-based bimetallic chalcogenides, Fe_x_A_y_B; A represents Ni, Co, Cr, or Mn, and B represents S, Se, or Te, and x and y represent the concentration of respective atoms. The stacked generalization model is trained on the dataset obtained using first-principles density functional theory. The model achieves MSE, MAE, and R^2^ values of 1.655 (µB)^2^, 0.546 (µB), and 0.922 respectively on an independent test set, indicating that our model predicts the compositional dependent magnetism in bimetallic chalcogenides with a high degree of accuracy. A generalized algorithm is also developed to test the universality of our proposed model for any concentration of Ni, Co, Cr, or Mn up to 62.5% in bimetallic chalcogenides.

## Introduction

Permanent magnets have been playing a great role in the development of science since their discovery^1^. They are used almost in every sector of current technology^2^. The growing awareness of green earth and renewable energy sources has also boosted the use of permanent magnets in energy sectors such as hydro-energy, wind, and wave energy as well as in electric vehicles. With the increased demand for permanent magnets, the rare-earth elements used in manufacturing strong permanent magnets are in a critical state of running out. Numerous experimental and theoretical research works have been done to develop new magnetic materials^3–12^.

For example, iron-based chalcogenides have been extensively studied, both theoretically and experimentally for their intriguing magnetic behavior^13–20^; ferromagnetism, ferrimagnetism, and antiferromagnetism are reported for different chalcogens^14,15,18^. Varying the elemental composition of the transition metal chalcogenides by changing both the metal elements as well as the chalcogens may reveal a new form of magnetic material. However, investigating all possible compositions of chalcogenides is an open challenge. The experimental investigation involves the synthesis and characterization of these materials, which are prohibitively expensive and time-consuming. Predictive calculation based on the first-principles density functional theory (DFT)^21–23^ that explicitly includes electron–electron interactions within an effective single-particle picture is also numerically challenging considering a multitude of compositional configurations of chalcogenides. In a situation like this, a well-established data-driven approach could offer a faster and computationally cost-effective alternative to those expensive and time-consuming experimental or computational methods. In recent years, much data-driven research has been performed to study magnetic properties^24–29^, band gaps^30,31^, as well as chemical properties^32,33^ of materials using machine learning models trained on DFT and experimental data. The catalytic activity of the complicated chemical system has been investigated using machine learning methods^32^. Also, the accurate predictions of band gaps in functionalized MXene exhibit the credibility of the machine learning approach^30^. Additionally, complex phenomena such as magnetic ordering, and magnetic moment have been successfully studied in 2d materials using a data-driven approach^24^. DFT-aided autonomous material search system has been designed to identify magnetic alloys^25^. Furthermore, the properties of rare-earth lean magnets are studied using DFT-aided machine learning^34^. In particular, the growing interest in studying the magnetic properties of materials using DFT-based machine learning models has highlighted the importance of DFT in the field of data-driven material science^22,25,29–32^.

In this work, considering the recent advances of artificial intelligence in the multidisciplinary field of science and technology^35–38^*,* we attempt to apply machine learning methods to develop a predictive tool that learns meaningful patterns from data and predicts the compositional dependent magnetism in Fe-based bimetallic chalcogenides Fe_x_A_y_B; A represents Ni, Co, Cr, or Mn, and B represents S, Se, or Te, and x and y are the concentration of respective elements. In order to develop a machine learning-based approach for identifying magnetism in Fe-based bimetallic chalcogenides, we generate a dataset of structures representing 4348 compositional configurations of Fe-based bimetallic chalcogenides Fe_x_A_y_B using density functional theory (DFT) calculations. We obtain magnetization of each compositional configuration using spin-polarized DFT calculation. This dataset is subsequently used to train the various ML algorithms such as Linear Regression, Support Vector Regressor^39,40^, Random Forest^41^, Decision Trees^42^, K-nearest neighbors^43^, Extreme Gradient Boosting^44,45^, and Artificial Neural Network^46,47^. Based on a tenfold cross-validation^48^ score, we selected the six best machine learning algorithms to develop an ensemble model based on stacked generalization for predicting magnetism in bimetallic chalcogenides. We obtained MSE, MAE, and R^2^ values of 1.655 (µB)^2^, 0.546 (µB), and 0.922 when we tested the final stacked model on an independent DFT test data.

## Materials and methods

Our approach to discovering magnetism in Fe-based bimetallic chalcogenides is based on a supervised machine-learning approach. Initially, we generated a dataset of 4348 structures representing various compositional configurations and then performed DFT^21^ calculations and obtained magnetization in the unit cell. Then, we performed feature engineering where we employed a set of descriptors (features) that are suitable for describing magnetization in the chalcogenides. We divided the dataset into training and test sets. Subsequently, we trained the model using cross-validation and grid search methods to determine the best-performing model. Finally, we tested the performance of our proposed model on the (independent) test set. Each of these four stages is briefly discussed in the following subsections.

### Dataset

**Figure 1:**
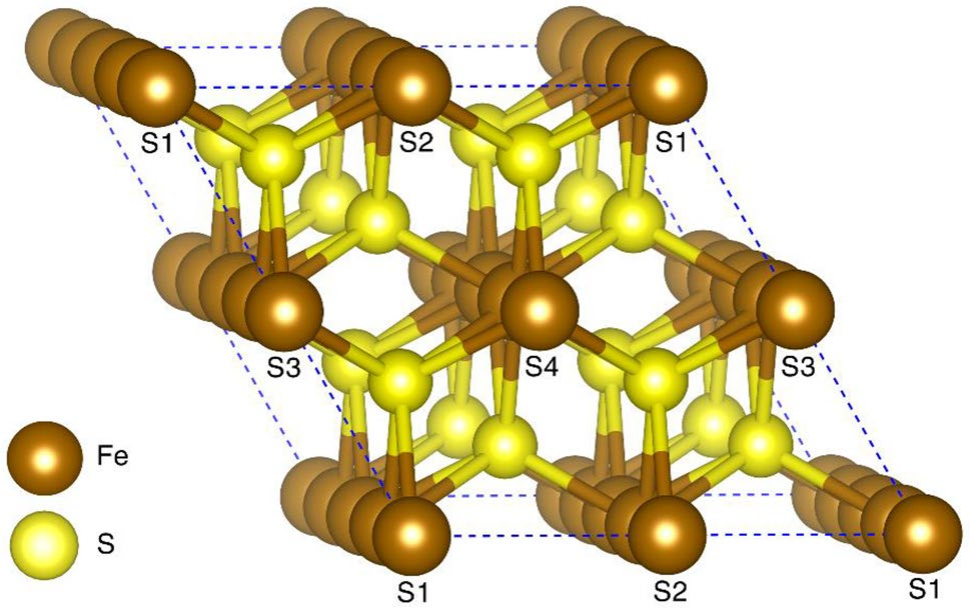
Unit cell of hexagonal FeS. S1, S2, S3, and S4 represent the substitutional sites for the transition metal elements.

To create the dataset, we employed the first principles DFT^21^ calculations. We started with constructing a primitive cell of hexagonal (space group p63/mmc) Iron-Sulfide (FeS) consisting of two Fe atoms and two S atoms. It has been reported that the chalcogenide can be easily synthesized in the hexagonal form as compared to the tetragonal structure^49^. Vienna ab initio simulation package (VASP)^50,51^ is used for the DFT calculations; a plane wave basis with a cutoff energy of 720 eV is used. The atomic structure in the unit cell is optimized without symmetry constraint until the residual force on each atom is smaller than 0.001 eV/ Å. The convergence criterion for the total energy is set at 10^−10^ eV. The exchange and correlation are approximated using a gradient-corrected Perdew–Burke–Ernzerhof (PBE)^52^ exchange–correlation functional and the electron–ion interactions are treated with the Projector Augmented Wave (PAW)^53^ potential. A Monkhrost-Pack scheme with a 3 × 3 × 3 K-point grid is used to sample the first Brillouin zone in the reciprocal lattice. Using the optimized lattice parameters, we expanded the primitive cell of hexagonal FeS to a bigger unit cell which consists of 16 Fe-atoms and 16 S-atoms as shown in Fig. 1.

Subsequently, we used a substitution technique to create bimetallic chalcogenides (Fe_x_A_y_B) of different atomic compositions; x and y represent the respective concentration of elements. Substitution technique^24,54^ is a common practice in material science to create a defect as well as new material. The Fe atoms in the structures were substituted by A (Ni, Co, Cr, or Mn) and S atoms by B (Se or Te). To describe the local geometry of the structure, we designated four atomic sites by S1, S2, S3, and S4 as shown in Fig. 1. A number was assigned to these sites S1, S2, S3, and S4 depending upon how many Fe-atoms are replaced at those atomic sites keeping the chalcogen concentration unchanged. For example, when we substitute two Fe-atoms at site S1, one Fe atom at site S2, and no substitution at S3 and S4, then S1 = 2, S2 = 1, and S3 = S4 = 0. This leads to x = (16–3)/16 and y = 3/16. Based on the number and site of substitutions, we generated 4348 bimetallic chalcogenide structures of different compositions. Subsequently, spin-polarized DFT calculations were performed to calculate magnetization for each of these compositions to obtain the data set to develop the machine learning models.

Once the dataset is created, the next step is to identify/define the descriptors or features for the problem as the choice of descriptors is one of the most important aspects of any machine learning-based approach. We choose 12 descriptors for our problem where the 8 descriptors describe the concentration of metal elements such as metal Fe, Ni, Co, Cr, Mn, and chalcogens elements S, Se, and Te. For example, in Fe_0.6875_Ni_0.3125_Se, the eight descriptors are 0.6875, 0.3125, 0, 0, 0, 1, 0, 0 representing the concentration of each element for possible bimetallic chalcogenide configurations (please see the data table in the provided GitHub link). Four descriptors describe the location of substitutional sites S1, S2, S3, and S4. The choice of these descriptors was motivated by the fact that magnetic ordering in the substituted chalcogenides is dependent upon the substitutional sites too. The calculated magnetic moment of the unit cell obtained from the DFT is taken as the target variable and these 12 descriptors are the input for the supervised machine learning framework. To understand the correlations between the features, a correlation matrix is generated (see Fig. S2 in Supplementary Information). We observed a low level of correlation between the features.

The data were randomly split into a training set and a test set in the proportion of 85:15. Subsequently, both the training and test data set are normalized separately to avoid information leakage from the test set to the training set. The size of the training and test dataset is given in Table 1.

**Table 1:** Number of DFT-data points used for training and testing the machine learning models.

Dataset	Number of samples
Training data	3695
Independent testing data	653
Total data	4348

### Algorithm training and model selection

We trained seven different machine learning algorithms: Linear Regression (LR)^55^, Support Vector Regressor (SVR)^39,40^, Random Forests (RF)^41^, Decision Trees (DT)^43^, K-Nearest Neighbours (KNN)^43^, Extreme Gradient Boosting (XGBoost)^56^, and Artificial Neural Network (ANN). We used scikit-learn^57^ and TensorFlow Keras API^58^ to implement these models. To find the optimal hyperparameters for our model, we extensively performed tenfold cross-validation on the training set and grid search on different combinations of hyperparameters (See Table S1 in Supplementary Material). The description of algorithms and cross-validation techniques are briefly described in the following subsections.

We started with LR^55^, a popular machine-learning model that provides the best linear fit to the data points by finding a linear relationship between features and target output by minimizing the distance between the target value and the predicted value that lies on the best-fit line. The basic LR model takes the form: $y ={W}^{T}X$, where, y is the target, X = (1, x_1_, x_2_, …, x_n_) is the input feature vector, W = (w_0_, w_1_, w_2_, …, w_n_) is the weight vector.

**Figure 2:**
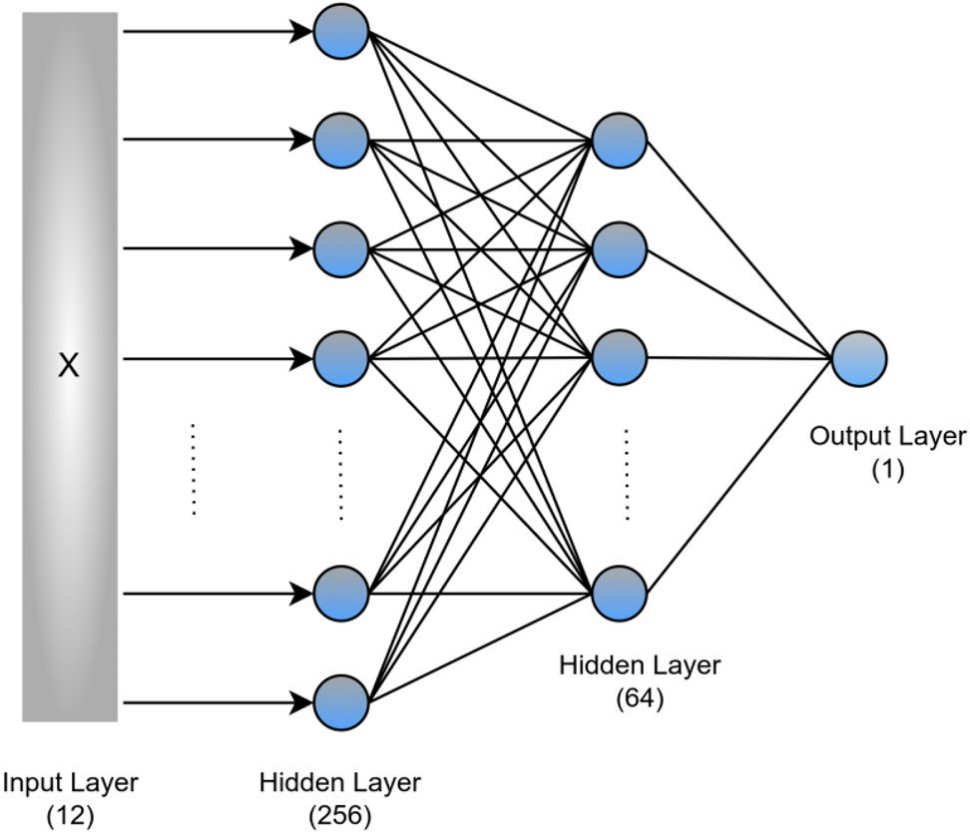
The architecture of an artificial neural network with two hidden layers with 256 and 64 neurons respectively.

For nonlinear relationships between features and the target, other algorithms such as DT, RF, KNN, SVR, XGBoost, and ANN offer better performance. DT^42^ splits the training examples into a tree-like structure based on the significant splitter in the input features. The splitting results in various leaf nodes, each of which represents a different prediction. RF^41,59^, which is also known for its capability in solving nonlinear problems, uses an ensemble learning approach that relies on the output of multiple decision trees. Thus, RF is a more powerful estimator as compared to DT and is less prone to overfitting and bias. The K-Nearest Neighbor (KNN)^43^ is a supervised algorithm that estimates the association between features and target variables based on the average output of the other nearest K data points. In our experiment, we set the number of nearest neighbors to K = 5. SVM^39^ uses the kernel functions that transform the low dimensional data into a higher dimensional feature space such that it can find a separation hyperplane that maximizes the margin between different classes. For regression problems, SVR^40^ fits the best hyperplane on training data to predict the discrete values. We have used the XGBoost^56^ which is a gradient-boosting-based decision tree algorithm. It uses a gradient descent approach to minimize the loss and combines different models using an ensemble approach. XGBoost and RF have nearly similar model representations with different training algorithms. XGBoost is based on serialized base learners, whereas RF is based on parallelized base learners. We have also implemented an artificial neural network (ANN) with two hidden layers using a simple feed-forward neural network architecture that learns by comparing initial outputs with the provided target by adjusting weights and biases through backpropagation. The architecture of the ANN-based model is shown in Fig. 2. After hyperparameter tuning, we found the best-performing neural network consists of two hidden layers having 256 and 64 neurons respectively. The details of hyperparameters are given in Table S1 (Supplementary Material). We have also analyzed how the features are contributing to a model prediction. Based on these calculations, which are performed using the random forest model, the concentrations of elements such as Mn, Cr, Te, and S are found to be the most dominant features as shown in Fig. S3 (Supplementary Information).

#### K-fold cross validation

To search for the best hyperparameters and to compare the performance of different models, we implemented a K-fold cross-validation algorithm on the training data. The K-fold cross-validation relies on a data partitioning scheme to ensure that the model can generalize the pattern on a diverse dataset. In this method, the dataset is randomly divided into K different sets. Following this, the model is trained using K-1 sets of the dataset and tested against the remaining set. The process is repeated K times and the results are statistically analyzed to choose the best-performing model. In this work, each model is trained and fine-tuned using K = 10 through a grid search process.

#### Stacked generalization

**Figure 3:**
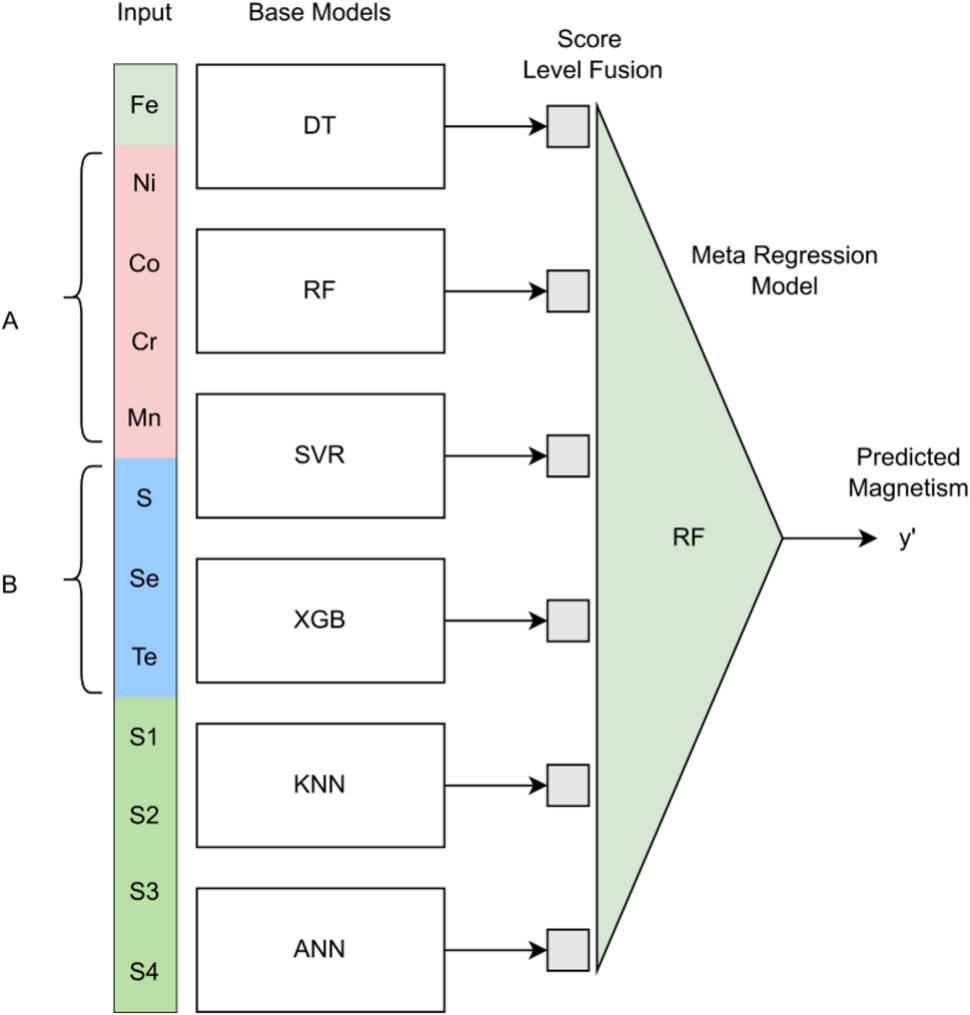
Block diagram for stack generalization.

After examining the individual models, we combined the best-performing models using a stacked generalization^60^ algorithm to improve the predictive performance. Stacked generalization is an ensemble approach that combines two or more pre-trained models (base learners) followed by a second-level regression model (meta learner). In this method, we stacked six base learners (DT, RF, SVR, XGB, KNN, and ANN) followed by a meta learner (RF) as shown in Fig. 3. It is noteworthy to point out that LR was omitted from the stacked generalization as its performance was not satisfactory.

Despite being a powerful technique that relies on the strength of multiple models, the stacked generalization approach is more prone to data leakage while performing cross-validation because the same dataset is used to train both the base models and meta-regression models resulting in model overfitting. The data leakage and overfitting in the cross-validation stage may mislead the model selection process. Hence, in this work, we implemented a stacking algorithm with cross-validation proposed by Wolpert^60^ to prevent data leakage and overfitting. In this technique, initially, the data is randomly divided into K sets. In the first stage, the base models are trained using K-1 sets and score-level features are extracted from those base models using the remaining set of training data. The process is repeated K times and each time a new dataset is prepared using the score-level features of the base models. In the second stage, a meta-regression model is trained using the data constructed from the first stage. Finally, each base model is trained using the entire training dataset and stacked together, which is subsequently connected to the previously trained meta-regression model (RF) to form the final model. It should be noted that in this work, each base learner and meta learner are trained and evaluated independently using the tenfold cross-validation method.

### Performance evaluation

To evaluate the performance of different machine learning regression models, we use three evaluation metrics: mean square error (MSE), mean absolute error (MAE), and coefficient of determination (R^2^), which are defined below.

$${\text{Mean Squared Error }}\left( {{\text{MSE}}} \right){ } = { }\frac{1}{n}\mathop \sum \limits_{i = 1}^{n} \left( {y_{i} - \hat{y}_{i} } \right)^{2}$$

$${\text{Mean Absolute Error }}\left( {{\text{MAE}}} \right){ } = { }\frac{1}{n}\mathop \sum \limits_{i = 1}^{n} \left( {y_{i} - \hat{y}_{i} } \right)$$

$${\text{Coefficient of determination }}\left( {R^{2} } \right) = 1 - \frac{{\mathop \sum \nolimits_{i}^{n} \left( {y_{i} - \hat{y}_{i} } \right)^{2} }}{{\mathop \sum \nolimits_{i}^{n} \left( {y_{i} - \overline{y}_{i} } \right)^{2} }}$$

where, $y_{i}$, $\hat{y}_{i}$ and $\overline{y}$ are the true, predicted, and the average value of y respectively.

MSE measures the average of the squares of residuals, while MAE measures the average of the residuals. They both have positive values; a smaller value indicates less error and better performance. MSE penalizes the model with larger errors than the MAE and hence is more sensitive to the outliers in the data. The lower the values of MSE and MAE are, the better the predictive performance of a model. The R^2^ score, also known as the coefficient of determination, is also a statistical measure in a regression model that represents the proportion of the variance in the dependent variable that is predictable from the independent variable(s). Its value lies between 0 and 1. Since R^2^ alone does not measure the accuracy of the predictions^61,62^, we have used this metric in conjunction with MSE and MAE to measure the performance of the regression models used in our study.

## Results and discussion

**Figure 4:**
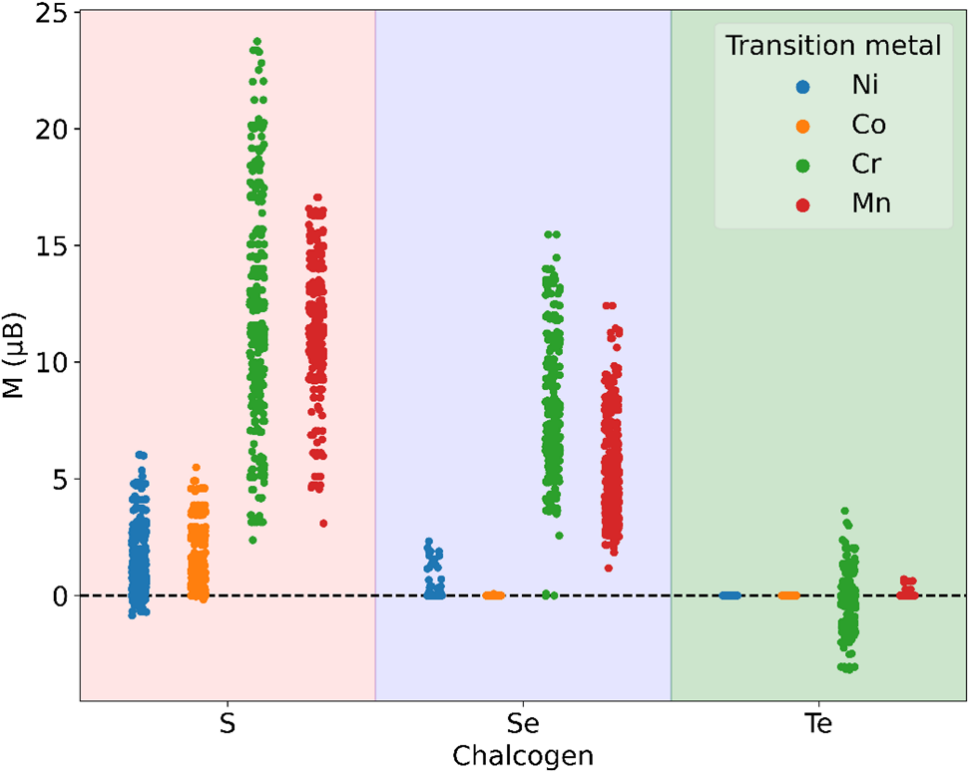
Dot plot showing the magnetic moment of Fe-based bimetallic chalcogenides; Fe_x_A_y_B where A represents Ni, Co, Cr, or Mn, and B represents S, Se, or Te, and x and y represent the concentration of respective atoms. Three shaded regions differentiate the magnetic moments of the chalcogenides containing S, Se, and Te respectively. Blue, orange, green, and red color dots correspond to the value of magnetic moments of the chalcogenides containing transition metal Ni, Co, Cr, and Mn respectively. Bimetallic chalcogenide with Cr and S exhibits a higher range for the magnetic moments.

First, we performed some exploratory data analysis on the DFT dataset. It reveals that the bimetallic chalcogenides containing S and Se are found to have higher magnetic moments compared to those containing Te as shown in Fig. 4. This fact is supported by earlier research^49^ that revealed FeS and FeSe exhibiting stronger magnetization than FeTe. Furthermore, we found that Fe-chalcogenides containing Cr and S have higher magnetic moments than those containing other transition metal elements (Ni, Co, or Mn) and chalcogen elements (Se and Te) as shown in Fig. 4. An increase in the magnetic moment is also noticeable when Cr or Mn concentration increases in chalcogenides containing S or Se (see Fig. S4 in Supplementary Information).

Further, the substitutional sites of transition metal elements in the chalcogenides are found to influence the target value (magnetic moment).

### Tenfold cross-validation results of base learners and meta learners

Table 2 shows the performance comparison of various machine learning models on the training dataset mentioned in Table 1. The RF model is found to perform well based on the comparison of mean MSE, mean MAE, and mean R^2^. The detailed calculation of performance measures is provided in Tables S2, S3 and S4 (Supplementary Information). Except for the LR approach, all models perform reasonably well, which could be understood from the plausible nonlinear relationship between the target variable and the features in our dataset. The well-performing models are subsequently used to develop the final stacked model.

**Table 2:** 10-Fold cross-validation results of different machine learning models.

Model name	Mean MSE(SD)	Mean MAE(SD)	Mean R^2^(SD)
Linear regression (LR)	7.00 (0.48)	2.16 (0.07)	0.69(0.02)
Decision tree (DT)	1.67 (0.32)	**0.54**(0.05)	0.93(0.01)
Random forest (RF)	**1.44** (0.21)	0.55(0.04)	0.93(0.01)
Support vector regressor (SVR)	1.62 (0.18)	0.57(0.03)	**0.92**(0.01)
Extreme gradient boosting (XGB)	1.58 (0.21)	0.68(0.04)	0.93(0.01)
K-nearest neighbour (KNN)	1.74 (0.30)	0.63(0.04)	**0.92**(0.01)
Artificial neural network (ANN)	1.45 (0.19)	0.59(0.04)	0.93(0.01)

The best value in each column is highlighted in bold.

Next, to find the best meta-regression model for the stacked generalization approach, we used the output from the individual base models to train LR, RF, and XGB and recalculated mean MSE, mean MAE, and mean R^2^. The results are presented in Table 3. RF model is found to be the best meta-learner.

**Table 3:** 10-Fold cross-validation results of various meta-learners.

Model name	Mean MSE(SD)	Mean MAE(SD)	Mean R^2^(SD)
Linear regression (LR)	1.40 (0.19)	0.56 (0.04)	0.94(0.01)
Extreme gradient boosting (XGB)	1.36 (0.27)	0.54(0.04)	0.94(0.01)
Random forest (RF)	**1.29** (0.28)	**0.50** (0.05)	**0.94** (0.01)

Significant values are in bold.

### Evaluation of the final stacked model on an independent (DFT) test dataset

Finally, we trained the models using the entire training dataset and tested them on the independent test dataset. Upon testing against the independent 653 DFT-test data points (Table 1), the MSE, MAE, and R^2^ of the stacked model are found to be 1.655 (µB)^2^, 0.546 µB, and 0.922 respectively compared to tenfold cross-validation values of 1.29 (µB)^2^, 0.50 µB and 0.94. These results show that our final model performs equally well on the test data as it did during the validation indicating the generalizability of the approach.

**Figure 5:**
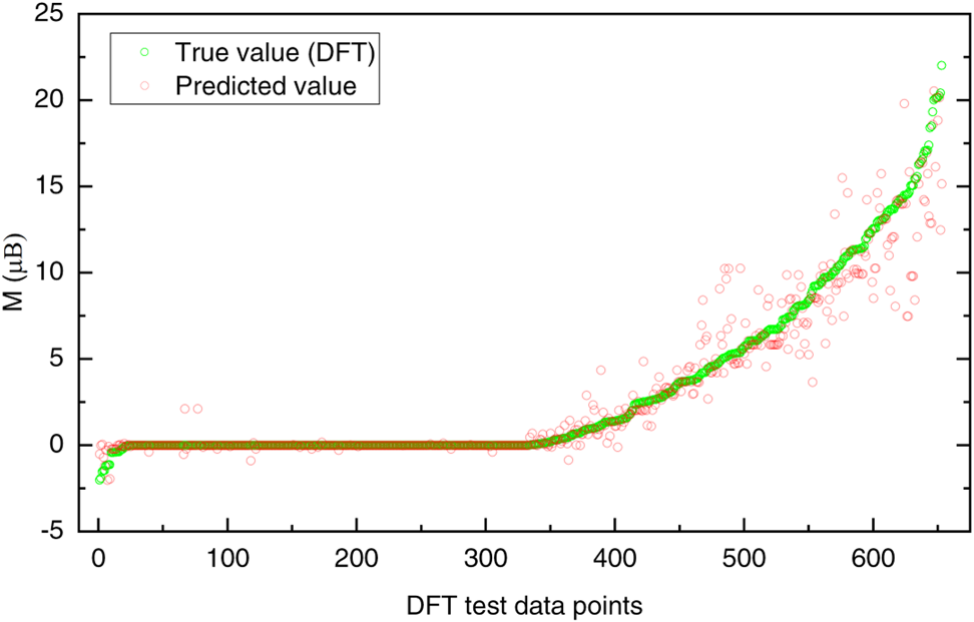
Scatter plots showing true (green circle) versus predicted (red circle) magnetic moments (M) in bimetallic chalcogenides. Data points from the independent test dataset, sorted in ascending order based on the value of the magnetic moment (M) obtained from DFT (true value).

Figure 5 shows the comparison between the true magnetization and predicted magnetization for each data point on the independent DFT test dataset. We have sorted the test data points in ascending order based on the value of the magnetic moment obtained from the DFT (true value). One can notice that the predictive performance of the model is much better for M < 14 μB. This is expected as only 3% of the entire training dataset is available to train the machine learning model for M > 14 μB. Though we noticed over and under prediction of magnetic moments in some instances, our model identifies non-magnetic and magnetic chalcogenides with a high degree of certainty. A deeper analysis of the under and over-predicted region reveal that a limited number of data points having similar target value are used during the training of the model, which explains the variation in the predicted target value (M) in those regions. Nevertheless, our study shows that the complex electronic interactions involved in the DFT calculations are well captured by the purposed model to predict magnetism in bimetallic chalcogenides.

### Expanding the applicability of the proposed model

In this work, a unit cell of FeS having 16 Fe and 16 S atoms is used to generate the DFT dataset. As a result, we have predefined fixed values of y (0.0625, 0.125, 0.1875, 0.25, 0.3125, 0.375, 0.4375, 0.5, 0.5625, 0.625) and *x *(= 1 *− y*) as multiples of 1/16. Furthermore, the descriptors S1, S2, S3, and S4 can each take integer values from 0 to 4. To overcome this limitation and increase the flexibility of our model, we have developed a generalized algorithm that can take the concentration y (or x) in percentages with a constraint of *0* < *y* < 0.625. It also allows the user to choose the concentration of substituted atoms on the atomic sites S1, S2, S3, and S4 in percentages. Then, the algorithm calculates the input features y, x, S1, S2, S3, and S4 in the suitable form required to feed the ML model. The detailed procedure is explained in Algorithm 1. The implementation of the algorithm is available in the provided GitHub link.
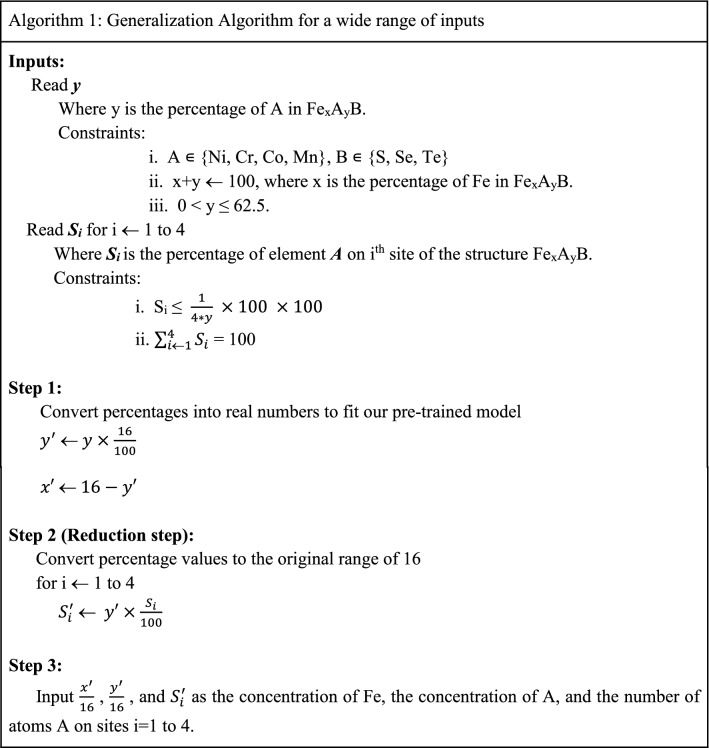


## Conclusions

The quest for new magnetic materials that are cheaper than the rare-earth element-based magnets is of significant interest in current years for their application that ranges from data storage to automotive vehicles to biomedical fields to the green energy sector. Experimental and computational investigation of possible alternative magnetic materials is expensive and time-consuming. In this work, we have used a data-driven framework that would accelerate the discovery of new magnetic materials. We used an optimized FeS structure and employed a substitution technique to design new bimetallic chalcogenides of different compositions. The first principle DFT is used to generate training and test data. After training and evaluating several machine learning models, we have developed a stacked machine learning model combining several best-performing base models as the final predictive tool. The final model has shown a high degree of accuracy on the independent DFT-test data with MSE, MAE, and R^2^ values of 1.655 (µB)^2^, 0.546 µB, and 0.922 respectively. Additionally, we have developed a generalized algorithm to expand the applicability of our model to a wide range of inputs. The predicted data reveal the Fe-based bimetallic chalcogenides containing chalcogen element S and a higher concentration of transition metal Cr yielding higher magnetic moments than the other configurations, which is consistent with the DFT data. This work presents a strategy for discovering a new magnetic material made from less expensive and more abundant elements that would eventually replace the costly existing magnetic materials made out of rare-earth metals.

## Supplementary Information

Supplementary Information.

## Data Availability

The trained model, training (DFT) and test (DFT) dataset, predicted dataset, source code, and other relevant materials are publicly available at https://github.com/dppant/magnetism-prediction.

## Abbreviations

DFT: Density function theory
MSE: Mean squared error
MAE: Mean absolute error
ML: Machine learning
DT: Decision tree
RF: Random forest
SVM: Support vector machine
SVR: Support vector regressor
ANN: Artificial neural network
KNN: K-nearest neighbour
XGB: Extreme gradient boosting
LR: Linear regression
SD: Standard deviation
API: Application program interface
PBE: Perdew–Burke–Ernzerhof
PAW: Projector augmented wave
VASP: Vienna ab initio simulation package

## References

[1] Coey JMD (2001). Magnetism and Magnetic Materials.

[2] Thompson DA, Best JS (2000). The future of magnetic data storage techology. IBM J. Res. Dev..

[3] Balasubramanian B, Das B, Skomski R, Zhang WY, Sellmyer DJ (2013). Novel nanostructured rare-earth-free magnetic materials with high energy products. Adv. Mater..

[4] Kuz’min MD, Skokov KP, Jian H, Radulov I, Gutfleisch O (2014). Towards high-performance permanent magnets without rare earths. J. Phys. Condens. Matter.

[5] Lottini E (2016). Strongly exchange coupled core|shell nanoparticles with high magnetic anisotropy: A strategy toward rare-earth-free permanent magnets. Chem. Mater..

[6] Anagnostopoulou E (2016). Dense arrays of cobalt nanorods as rare-earth free permanent magnets. Nanoscale.

[7] Balamurugan B, Das B, Zhang WY, Skomski R, Sellmyer DJ (2014). Hf–Co and Zr–Co alloys for rare-earth-free permanent magnets. J. Phys. Condens. Matter.

[8] Gao TR (2013). Combinatorial exploration of rare-earth-free permanent magnets: Magnetic and microstructural properties of Fe–Co–W thin films. Appl. Phys. Lett..

[9] Pant D, Aryal S, Mandal S, Pati R (2021). Emergence of ferromagnetism due to spontaneous symmetry breaking in a twisted bilayer graphene nanoflex. Nano Lett..

[10] Vishina A (2020). High-throughput and data-mining approach to predict new rare-earth free permanent magnets. Phys. Rev. B.

[11] Gao Q, Opahle I, Gutfleisch O, Zhang H (2020). Designing rare-earth free permanent magnets in Heusler alloys via interstitial doping. Acta Mater..

[12] Pant D, Pati R (2022). Phase transition from a nonmagnetic to a ferromagnetic state in a twisted bilayer graphene nanoflake: The role of electronic pressure on the magic-twist. Nanoscale.

[13] Liu A, Chen X, Zhang Z, Jiang Y, Shi C (2006). Selective synthesis and magnetic properties of FeSe_2_ and FeTe_2_ nanocrystallites obtained through a hydrothermal co-reduction route. Solid State Commun..

[14] Kang L (2020). Phase-controllable growth of ultrathin 2D magnetic FeTe crystals. Nat. Commun..

[15] Deng Y (2018). Gate-tunable room-temperature ferromagnetism in two-dimensional Fe_3_GeTe_2_. Nature.

[16] Oyler KD, Ke X, Sines IT, Schiffer P, Schaak RE (2009). Chemical synthesis of two-dimensional iron chalcogenide nanosheets: FeSe, FeTe, Fe(Se, Te), and FeTe_2_. Chem. Mater..

[17] Matthews PD, Akhtar M, Malik MA, Revaprasadu N, O’Brien P (2016). Synthetic routes to iron chalcogenide nanoparticles and thin films. Dalton Trans..

[18] Nakamura K, Ito T, Freeman AJ (2005). Half-metallic ferrimagnetism in zincblende Mn-doped transition metal chalcogenides. Phys. Rev. B.

[19] Dong C (2011). Revised phase diagram for the FeTe_1__−__x_Se_x_ system with fewer excess Fe atoms. Phys. Rev. B.

[20] Böhmer AE (2015). Origin of the tetragonal-to-orthorhombic phase transition in FeSe: A combined thermodynamic and NMR study of nematicity. Phys. Rev. Lett..

[21] Parr RG, Yang W (1994). Density-Functional Theory of Atoms and Molecules.

[22] Hohenberg P, Kohn W (1964). Inhomogeneous electron gas. Phys. Rev..

[23] Kohn W, Sham LJ (1965). Self-consistent equations including exchange and correlation effects. Phys. Rev..

[24] Rhone TD, Chen W, Desai S (2020). Data-driven studies of magnetic two-dimensional materials. Sci. Rep..

[25] Iwasaki Y, Sawada R, Saitoh E (2021). Machine learning autonomous identification of magnetic alloys beyond the Slater-Pauling limit. Commun. Mater..

[26] Kusne AG (2015). On-the-fly machine-learning for high-throughput experiments: Search for rare-earth-free permanent magnets. Sci. Rep..

[27] Halder A, Ghosh A, Dasgupta TS (2019). Machine-learning-assisted prediction of magnetic double perovskites. Phys. Rev. Mater..

[28] Möller JJ, Körner W, Krugel G, Urban DF, Elsässer C (2018). Compositional optimization of hard-magnetic phases with machine-learning models. Acta Mater..

[29] Merker HA (2022). Machine learning magnetism classifiers from atomic coordinates. iScience.

[30] Rajan AC (2018). Machine-learning-assisted accurate band gap predictions of functionalized MXene. Chem. Mater..

[31] Zhuo Y, Mansouri Tehrani A, Brgoch J (2018). Predicting the band gaps of inorganic solids by machine learning. J. Phys. Chem. Lett..

[32] Wan X, Zhang Z, Yu W, Guo Y (2021). A density-functional-theory-based and machine-learning-accelerated hybrid method for intricate system catalysis. Mater. Rep. Energy.

[33] Jinnouchi R, Asahi R (2017). Predicting catalytic activity of nanoparticles by a DFT-aided machine-learning algorithm. J. Phys. Chem. Lett..

[34] Halder A, Rom S, Ghosh A, Saha-Dasgupta T (2020). Prediction of the properties of the rare-earth magnets Ce_2_ Fe_17__−__x_ Co_x_ CN: A combined machine-learning and Ab Initio study. Phys. Rev. Appl..

[35] Schleder GR, Padilha ACM, Acosta CM, Costa M, Fazzio A (2019). From DFT to machine learning: Recent approaches to materials science–a review. J. Phys. Mater..

[36] Butler KT, Davies DW, Cartwright H, Isayev O, Walsh A (2018). Machine learning for molecular and materials science. Nature.

[37] The rise of data-driven modeling. *Nat. Rev. Phys.* **3**, 383–383 (2021).

[38] Schmidt J, Marques MRG, Botti S, Marques MAL (2019). Recent advances and applications of machine learning in solid-state materials science. Npj Comput. Mater..

[39] Cortes C, Vapnik V (1995). Support-vector networks. Mach. Learn..

[40] Smola AJ, Schölkopf B (2004). A tutorial on support vector regression. Stat. Comput..

[41] Breiman L (2001). Random forests. Mach. Learn..

[42] Breiman L (1984). Classification and Regression Trees.

[43] Bentley JL (1975). Multidimensional binary search trees used for associative searching. Commun. ACM.

[44] Friedman JH (2001). Greedy function approximation: A gradient boosting machine. JSTOR.

[45] Hastie T, Tibshirani R, Friedman J (2013). The Elements of Statistical Learning: Data Mining, Inference, and Prediction.

[46] McCulloch WS, Pitts W (1943). A logical calculus of the ideas immanent in nervous activity. Bull. Math. Biophys..

[47] Specht DF (1991). A general regression neural network. IEEE Trans. Neural Netw..

[48] Gareth J, Daniela W, Trevor H, Robert T (2013). An Introduction to Statistical Learning.

[49] Parker DS (2017). Strong 3D and 1D magnetism in hexagonal Fe-chalcogenides FeS and FeSe vs. weak magnetism in hexagonal FeTe. Sci. Rep..

[50] Kresse G, Furthmüller J (1996). Efficiency of ab-initio total energy calculations for metals and semiconductors using a plane-wave basis set. Comput. Mater. Sci..

[51] Kresse G, Furthmüller J (1996). Efficient iterative schemes for ab initio total-energy calculations using a plane-wave basis set. Phys. Rev. B.

[52] Perdew JP, Burke K, Ernzerhof M (1996). Generalized gradient approximation made simple. Phys. Rev. Lett..

[53] Kresse G, Joubert D (1999). From ultrasoft pseudopotentials to the projector augmented-wave method. Phys. Rev. B.

[54] Picard CJ, Needs RJ (2011). Ab initio random structure searching. J. Phys. Condens. Matter.

[55] Bishop CM (2016). Pattern Recognition and Machine Learning.

[56] Chen, T. & Guestrin, C. XGBoost: A Scalable Tree Boosting System. In *KDD'16: Proceedings of the 22nd ACM SIGKDD International Conference on Knowledge Discovery and Data Mining*, pp. 785–794, New York, USA 10.1145/2939672.2939785 (2016).

[57] Pedregosa F, Varoquaux G, Gramfort A, Michel V (2011). Scikit-learn: Machine learning in Python. J. Mach. Learn. Res..

[58] TensorFlow Developers. *TensorFlow*. 10.5281/ZENODO.4724125 (2022).

[59] Geurts P, Ernst D, Wehenkel L (2006). Extremely randomized trees. Mach. Learn..

[60] Wolpert DH (1992). Stacked generalization. Neural Netw..

[61] Spanos A (2019). Probability Theory and Statistical Inference: Empirical Modeling with Observational Data.

[62] Hagquist C, Stenbeck M (1998). Goodness of fit in regression analysis: R2 and G2 reconsidered. Qual. Quant..

