# Supplementary material for: DFT-aided machine learning-based discovery of magnetism in Fe-based bimetallic chalcogenides
Source: Sci Rep. 2023 Feb 25;13:3277. doi: 10.1038/s41598-023-30438-w (PMC9968303; doi:10.1038/s41598-023-30438-w)
Supplement: Supplementary file 1 — Supplementary Information. [file 41598_2023_30438_MOESM1_ESM.docx]

**SUPPLEMENTARY INFORMATION**

**DFT-aided Machine Learning-based discovery of Magnetism in Fe-based Bimetallic Chalcogenides**

Dharmendra Pant^1^, Suresh Pokharel^2^, Subhasish Mandal^3^, Dukka B KC^2^ and Ranjit Pati^1,4^*

^1^ Department of Physics, Michigan Technological University, Houghton, MI 49931, USA

^2^ Department of Computer Science, Michigan Technological University, Houghton, MI 49931, USA

^3^ Department of Physics & Astronomy, West Virginia University, Morgantown, WV 26506, USA

^4^ Henes Center for Quantum Phenomena, Michigan Technological University, Houghton, Michigan 49931, USA.

*Corresponding author. Email: patir@mtu.edu

Table S1: Hyperparameters of various machine learning models.

| **Name of Model** | **Hyperparameters** |
| --- | --- |
| Linear Regression (LR) | - |
| Decision Tree (DT) | - |
| Random Forest (RF) | n_estimators = 500, min_samples_split = 13 |
| Support Vector Regressor (SVR) | Regularization Parameter C = 12, Kernel = ‘rbf’, Gamma = 1 |
| Extreme Gradient Boosting (XGB) | Learning_rate = 0.1, n_estimators = 100, criterion='squared_error' |
| K-Nearest Neighbour (KNN) | k = 5 |
| Artificial Neural Network (ANN) | No. of hidden layers: **2**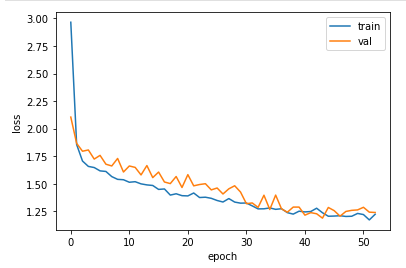  No. of neurons in hidden layer 1: **256**  No. of neurons in hidden layer 2: **64**  Initial learning rate: **0.01**  Optimizer: **adam**  Loss function: **mse**  Intermediate layer’s activation function: **ReLU**  Regularization Method: **Early Stopping**    **Figure S1:** The training and validation loss curve of ANN base model. |

Note: The names of hyperparameters are based on python’s scikit-learn library. Hyperparameters not mentioned in the table are taken as the default values provided by scikit-learn.

Table S2: 10-Fold Cross Validation Mean Squared Errors of Base and Stacked Models

| **FOLD** | **LR** | **DT** | **RF** | **SVR** | **XGB** | **KNN** | **ANN** | **Stack_RF** | **Stack_LR** | **Stack_XGB** |
| --- | --- | --- | --- | --- | --- | --- | --- | --- | --- | --- |
| 1 | 6.62715122 | 1.72058446 | 1.44315052 | 1.542734547 | 1.52222125 | 1.57284425 | 1.245126105 | 1.05055168 | 1.26941185 | 1.1359125 |
| 2 | 7.32733797 | 1.72237218 | 1.55489068 | 1.575030749 | 1.82667785 | 2.07397086 | 1.595029988 | 1.36438011 | 1.49634279 | 1.4740999 |
| 3 | 6.4675365 | 1.77819267 | 1.24913604 | 1.826050232 | 1.23848509 | 1.64270622 | 1.329177284 | 1.09870072 | 1.39430711 | 1.13183395 |
| 4 | 7.2682616 | 1.86422235 | 1.53390967 | 1.6299566 | 1.55040293 | 2.0523524 | 1.376909337 | 1.20741349 | 1.50210739 | 1.36071174 |
| 5 | 7.04722049 | 1.81037493 | 1.49640879 | 1.344262679 | 1.54734647 | 2.11677536 | 1.225007842 | 0.9135783 | 1.07019391 | 0.96411346 |
| 6 | 7.42613035 | 1.11439629 | 1.19264086 | 1.867063862 | 1.47612781 | 1.40599941 | 1.765705329 | 1.65054692 | 1.59395054 | 1.72165875 |
| 7 | 6.72947548 | 1.22354608 | 1.17735006 | 1.294449811 | 1.27367941 | 1.30825049 | 1.218063329 | 1.06149868 | 1.14348121 | 1.13442854 |
| 8 | 7.83228801 | 1.88503437 | 1.81151611 | 1.756135703 | 1.80597346 | 1.83604152 | 1.562212693 | 1.81457607 | 1.6772614 | 1.77843018 |
| 9 | 6.24320202 | 1.4067101 | 1.28081362 | 1.643312215 | 1.80597028 | 1.45116284 | 1.532521336 | 1.46419513 | 1.45186315 | 1.51824696 |
| 10 | 7.09400827 | 2.18679418 | 1.65060402 | 1.689829077 | 1.77264267 | 1.94825641 | 1.630666771 | 1.2913116 | 1.44306992 | 1.42675333 |
| **Mean** | **7.006261191** | **1.671222761** | **1.43904203** | **1.616882548** | **1.581952722** | **1.740835976** | **1.448042001** | **1.29167527** | **1.404198927** | **1.364618931** |
| **SD** | **0.4867740103** | **0.3274605895** | **0.21069218** | **0.1878113318** | **0.217711428** | **0.3022960303** | **0.1940895935** | **0.2859063031** | **0.191551171** | **0.2704047405** |

Table S3: 10-Fold Cross Validation Mean Absolute Errors of Base and Stacked Models

| **FOLD** | **LR** | **DT** | **RF** | **SVR** | **XGB** | **KNN** | **ANN** | **Stack_RF** | **Stack_LR** | **Stack_XGB** |
| --- | --- | --- | --- | --- | --- | --- | --- | --- | --- | --- |
| 1 | 2.09016338 | 0.56265445 | 0.54921349 | 0.5819318422 | 0.67384896 | 0.61521189 | 0.5721343137 | 0.46276702 | 0.55601135 | 0.51207738 |
| 2 | 2.19548632 | 0.52033796 | 0.53833581 | 0.5900867879 | 0.6963745 | 0.65325514 | 0.6324354788 | 0.53729156 | 0.58314077 | 0.56762393 |
| 3 | 2.09185641 | 0.55881602 | 0.52044547 | 0.5731176197 | 0.63253687 | 0.62359297 | 0.5538877578 | 0.46856493 | 0.54546457 | 0.50337995 |
| 4 | 2.20359408 | 0.53338759 | 0.55264502 | 0.6082833203 | 0.67211028 | 0.64534108 | 0.6065196503 | 0.52150743 | 0.63341657 | 0.59182881 |
| 5 | 2.13264152 | 0.57890467 | 0.57659403 | 0.5332364426 | 0.69701847 | 0.68876757 | 0.5302973962 | 0.44396747 | 0.50565994 | 0.47509169 |
| 6 | 2.26101853 | 0.49616773 | 0.54073683 | 0.5897179864 | 0.67943528 | 0.5984206 | 0.6529698412 | 0.58338632 | 0.5968483 | 0.61942878 |
| 7 | 2.1592235 | 0.4649394 | 0.4977806 | 0.5543679728 | 0.63676067 | 0.5579729 | 0.5589833685 | 0.44864079 | 0.52987258 | 0.51522122 |
| 8 | 2.26096425 | 0.62476902 | 0.62730201 | 0.5926486915 | 0.7305399 | 0.70775447 | 0.6001483426 | 0.56459545 | 0.59360688 | 0.58980287 |
| 9 | 2.06089983 | 0.52992519 | 0.537669 | 0.512713137 | 0.76143233 | 0.60454851 | 0.5422081675 | 0.46179715 | 0.51268689 | 0.51741879 |
| 10 | 2.22386409 | 0.61955251 | 0.58610792 | 0.6064516069 | 0.70871043 | 0.63049919 | 0.6133193189 | 0.51480413 | 0.59345139 | 0.5530049 |
| Mean | **2.167971191** | **0.548945454** | **0.552683018** | **0.5742555407** | **0.688876769** | **0.632536432** | **0.58629036** | **0.500732225** | **0.5650159** | **0.544487832** |
| SD | **0.07233571767** | **0.05075234021** | **0.0363570307** | **0.0315317648** | **0.039443866** | **0.04384838486** | **0.04080005** | **0.050336876** | **0.0416417** | **0.046742763** |

Table S4: 10-Fold Cross Validation R^2^ Score of Base and Stacked Models

| **FOLD** | **LR** | **DT** | **RF** | **SVR** | **XGB** | **KNN** | **ANN** | **Stack_RF** | **Stack_LR** | **Stack_XGB** |
| --- | --- | --- | --- | --- | --- | --- | --- | --- | --- | --- |
| 1 | 0.69673454 | 0.92134751 | 0.93470327 | 0.9304424805 | 0.93034154 | 0.92802498 | 0.9407629356 | 0.95755064 | 0.94754474 | 0.94788304 |
| 2 | 0.669495 | 0.92171383 | 0.93015196 | 0.9326469857 | 0.91760634 | 0.90645201 | 0.9256737052 | 0.94368298 | 0.94040755 | 0.93893776 |
| 3 | 0.67006262 | 0.91110731 | 0.93565524 | 0.9140209216 | 0.93681945 | 0.91619836 | 0.9450088001 | 0.95122794 | 0.94157593 | 0.94791067 |
| 4 | 0.70898963 | 0.93177209 | 0.9378796 | 0.9321160768 | 0.93792418 | 0.91782687 | 0.9397057917 | 0.94541579 | 0.93488232 | 0.94073357 |
| 5 | 0.68934177 | 0.922262 | 0.93383371 | 0.9330895133 | 0.93178929 | 0.90668751 | 0.9336265457 | 0.95286276 | 0.94869786 | 0.95122426 |
| 6 | 0.67455509 | 0.95505482 | 0.94834341 | 0.9194862347 | 0.93530974 | 0.93838307 | 0.9225697238 | 0.93470857 | 0.93223048 | 0.92362365 |
| 7 | 0.71770984 | 0.94899879 | 0.95022533 | 0.9243306177 | 0.94656816 | 0.9451211 | 0.9261986312 | 0.94566295 | 0.94217978 | 0.94552302 |
| 8 | 0.69653499 | 0.92896712 | 0.92991423 | 0.9271052871 | 0.93002686 | 0.92886187 | 0.9303642065 | 0.92702146 | 0.93150372 | 0.9272172 |
| 9 | 0.72465749 | 0.93718971 | 0.94322363 | 0.9200040666 | 0.92035171 | 0.9359997 | 0.9279003003 | 0.93510991 | 0.93314261 | 0.93044158 |
| 10 | 0.69402051 | 0.9058802 | 0.92906787 | 0.9223854689 | 0.92356941 | 0.9159676 | 0.9329619411 | 0.94409534 | 0.93663257 | 0.93759585 |
| Mean | **0.694210148** | **0.928429338** | **0.937299825** | **0.9255627653** | **0.931030668** | **0.923952307** | **0.9324772581** | **0.943733834** | **0.93887975** | **0.93910906** |
| SD | **0.01918763118** | **0.01549920657** | **0.00759621130** | **0.006571199051** | **0.0087732912** | **0.01336632875** | **0.0073694695** | **0.0092629349** | **0.00618720** | **0.00944966** |


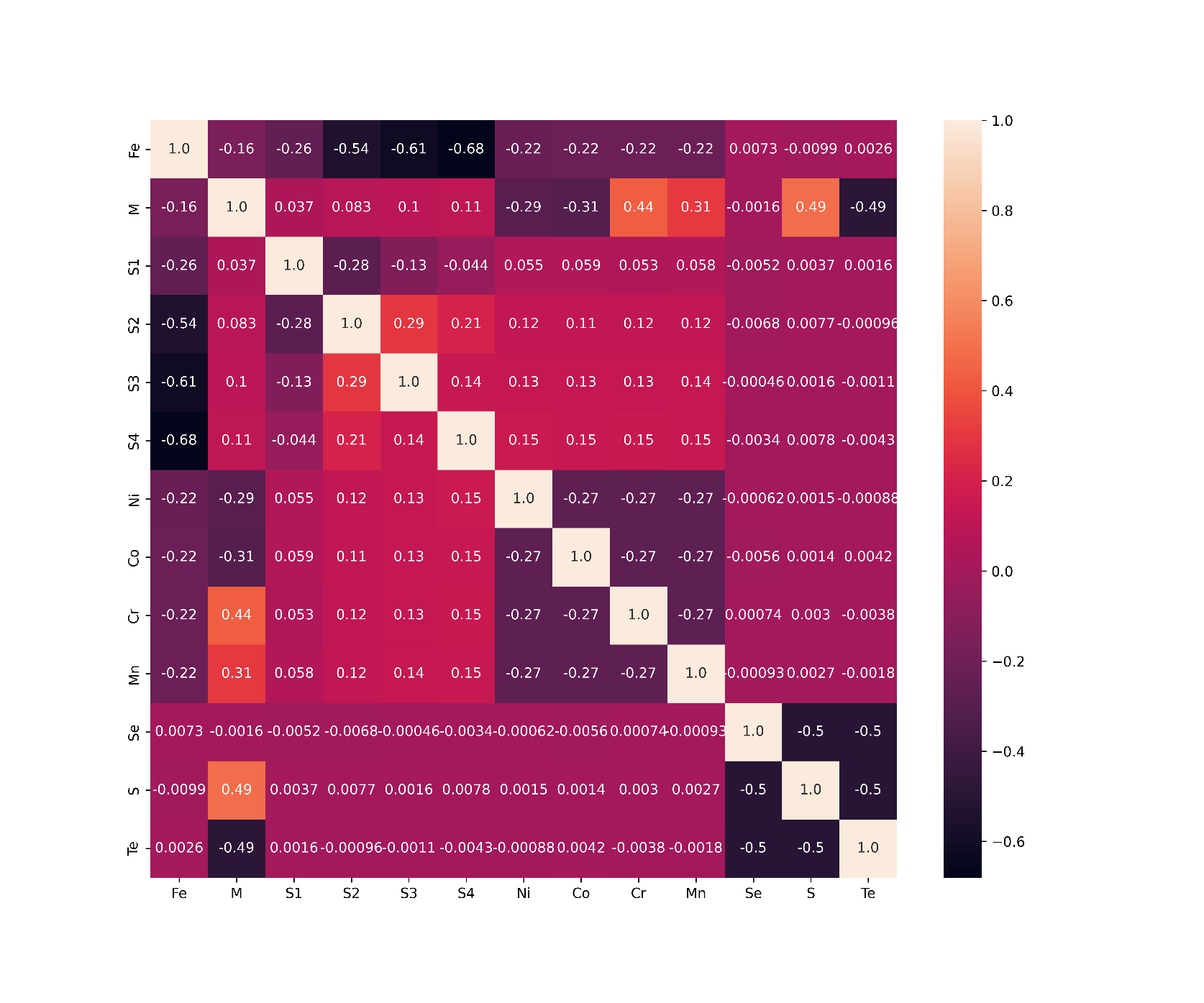


Figure S2. The correlation matrix shows the positive and negative correlations between features. A number inside a box measures the degree of the linear relationship between each pair of variables. The negative number indicates a negative correlation and the positive number indicates a positive correlation. We found a low level of correlation between the features.


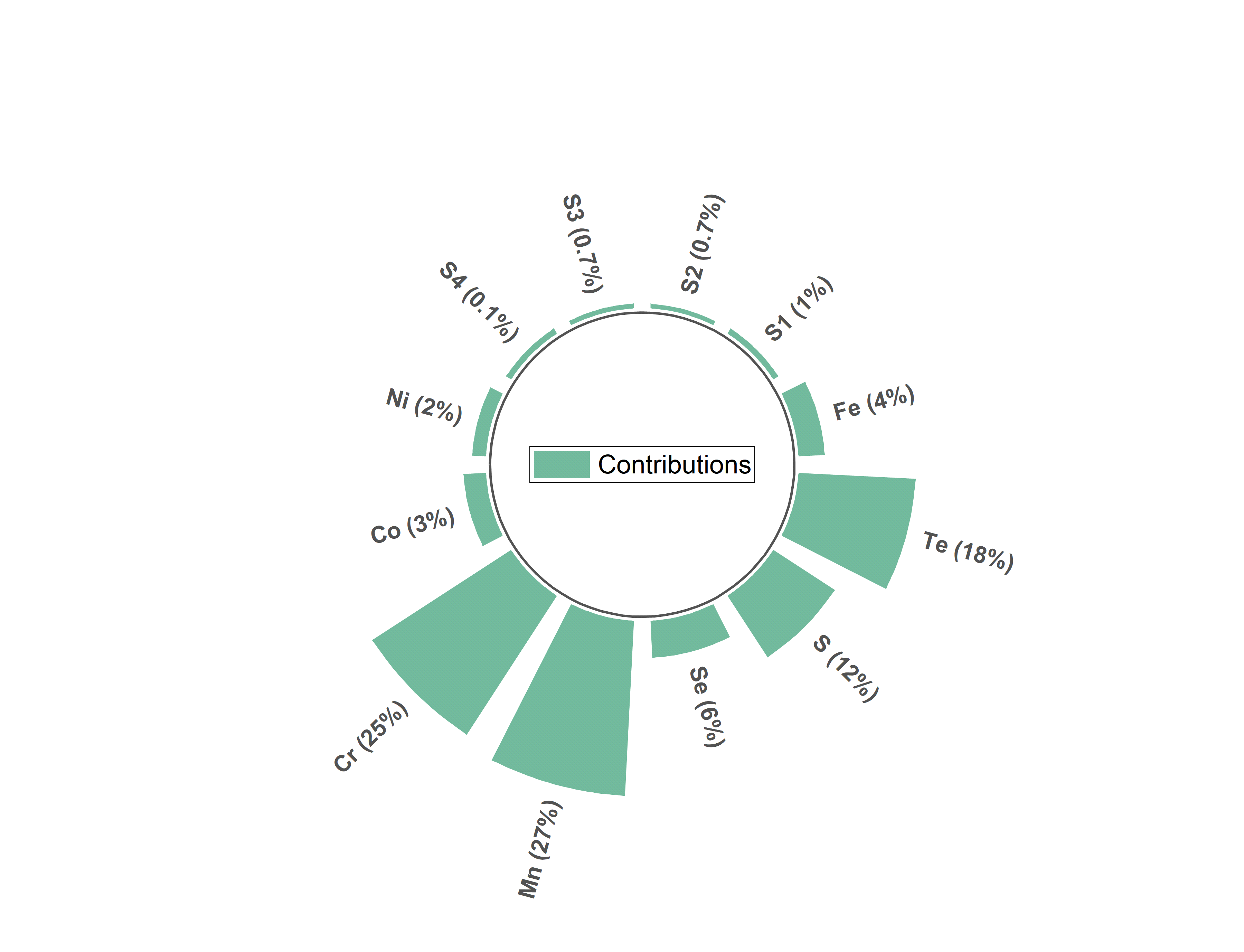


**Figure S3.** Radial bar plot showing the contribution of each feature to the model’s prediction. A random forest model is used to calculate the contribution of each feature. The concentrations of Mn, Cr, Te, and S are found to be the dominant features.


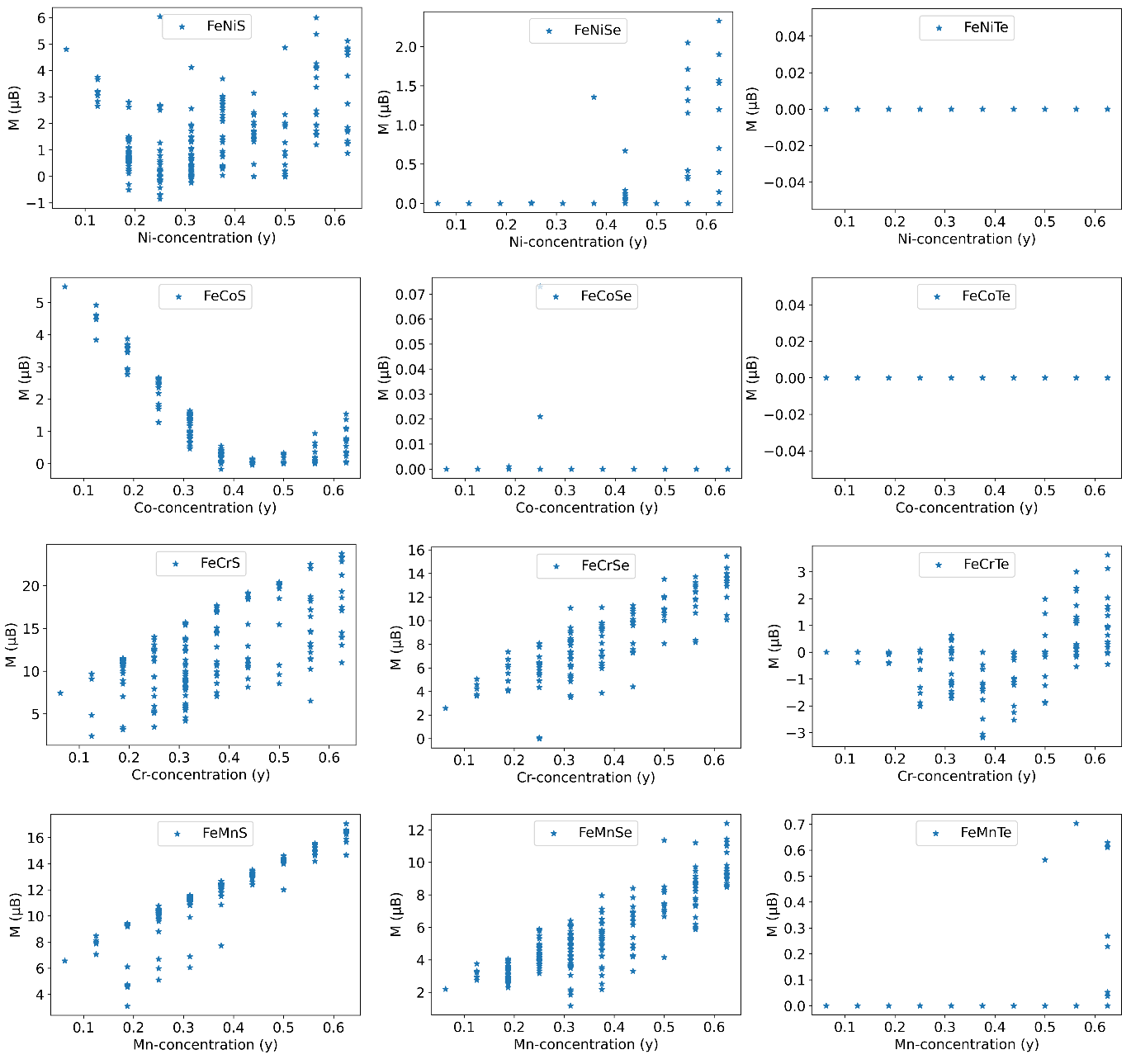


**Figure S4.** Magnetic moment in Fe-based bimetallic chalcogenides for various concentrations (y) of transition metal elements (DFT-data).
